# Development of the Screening Tool for Everyday Mobility and Symptoms (STEMS) for skeletal dysplasia

**DOI:** 10.1186/s13023-021-01681-z

**Published:** 2021-01-21

**Authors:** Penelope J. Ireland, Ravi Savarirayan, Tash Pocovi, Tracy Tate, Marie Coussens, Louise Tofts, Craig Munns, Verity Pacey

**Affiliations:** 1 Queensland Paediatric Rehabilitation Service, Queensland Children’s Hospital, 501 Stanley Street, South Brisbane, QLD 4101 Australia; 2 Department of Paediatrics, The University of Melbourne, Flemington Road, Parkville VIC, 3052 Australia; 3 Department of Health Professions, Macquarie University, Ground Floor, 75 Talavera Rd, Sydney, NSW 2109 Australia; 4 The Children’s Hospital At Westmead, Locked Bag 4001, Westmead, NSW 2145 Australia; 5 Department of Rehabilitation Sciences and Physiotherapy, Ghent University, Corneel Heymanslaan 10, 9000 Ghent, Belgium; 6 Kids Rehab, The Children’s Hospital, Locked Bag 4001, Westmead, NSW 2145 Australia; 7 Faculty of Medicine and Health Sciences, Macquarie University, Ground Floor, 75 Talavera Road, Sydney, NSW 2109 Australia; 8 Discipline of Child and Adolescent Health, University of Sydney, The Children’s Hospital At Westmead, Locked Bag 4001, Westmead, NSW Australia

**Keywords:** Skeletal dysplasia, Mobility, Pain, Fatigue, Achondroplasia, Osteogenesis imperfecta, Functional mobility tool

## Abstract

**Background:**

Skeletal dysplasia are genetic disorders of cartilage and bone, characterized by impairments commonly resulting in short stature, altered movement biomechanics, pain, fatigue and reduced functional performance. While current tools quantify functional mobility performance, they have not been standardly used in this population group and do not capture patient-reported symptoms such as pain or fatigue. This study evaluated a new tool, the Screening Tool for Everyday Mobility and Symptoms (STEMS), designed to accurately and objectively assess functional mobility and associated symptomology for individuals with skeletal dysplasia.

**Methods:**

Individuals aged 5–75 years with a skeletal dysplasia completed the STEMS, the Functional Mobility Scale (FMS) and Six Minute Walk Test (6MWT). The correlation among the STEMS, use of mobility aides, FMS and 6MWT normalised for leg length was calculated. One-way analysis of variance compared the STEMS symptomatology to normalised 6MWT distance.

**Results:**

One hundred and fifty individuals with skeletal dysplasia (76 achondroplasia, 42 osteogenesis imperfecta, 32 other; 74 < 18 years, 76 ≥ 18 years) participated. Almost two thirds of the group reported pain and/or fatigue when mobilising at home, at work or school and within the community, but only twenty percent recorded use of a mobility device. The STEMS setting category demonstrated highly significant correlations with the corresponding FMS category (r = − 0.983 to − 0.0994, all p < 0.001), and a low significant correlation with the normalised 6MWT distance (r = − 0.323 to − 0.394, all p < 0.001). A decreased normalised 6MWT distance was recorded for individuals who reported symptoms of pain and/or fatigue when mobilising at home or at work/school (all p ≤ 0.004). Those who reported pain only when mobilising in the community had a normal 6MWT distance (p = 0.43–0.46).

**Conclusions:**

The Screening Tool for Everyday Mobility and Symptoms (STEMS) is a useful new tool to identify and record mobility aide use and associated self-reported symptoms across three environmental settings for adults and children with skeletal dysplasia. The STEMS may assist clinicians to monitor individuals for changes in functional mobility and symptoms over time, identify individuals who are functioning poorly compared to peers and need further assessment, and to measure effectiveness of treatment interventions in both clinical and research settings.

## Introduction

Skeletal dysplasia occur in approximately 1 in every 5000 births, and incorporate over 350 individual disorders affecting cartilage and bone, the most common of which is achondroplasia [1]. There are several well recognised musculoskeletal impairments associated with skeletal dysplasia including short stature, macrocephaly, altered lower limb alignment, and scoliosis [2–4]. Disproportionate growth between the long bones and the underlying structures can lead to a number of orthopaedic, neurological, respiratory, ear, nose and throat and dental issues for individuals with skeletal dysplasia [3–5]. Furthermore, the complex interplay of the characteristic impairments of body structure can contribute directly and cyclically to activity limitations and participation restrictions for individuals with skeletal dysplasia across several areas of mobility, physical activity and day to day functioning [5, 6].

There is limited literature outlining the medical, health and social aspects of life for adolescents and adults with skeletal dysplasia, particularly in relation to their physical function and mobility. While some information is available regarding targeted profiles of motor skill development in children for varying forms of skeletal dysplasia such as achondroplasia [7–10] and osteogenesis imperfecta [11, 12], several research groups have highlighted issues with functional capacity post childhood. A significant number of adults with skeletal dysplasia develop physical limitations, pain and fatigue which impacts on their quality of life [13–17]. Recent studies have more specifically considered the relationship between pain and function and noted that chronic pain is prevalent in individuals with skeletal dysplasia, and associated with poor physical function [18, 19]. However, the specific impact that pain and/or fatigue has on mobility remains unclear.

The complex relationship existing between functional mobility performance and associated symptoms such as pain and fatigue for individuals with varying forms of proportionate and disproportionate short stature is currently not accurately reflected through use of existing objective clinical assessments or patient-reported questionnaires. While tools such as the Functional Mobility Scale (FMS) [20] and the Bleck Score [21] objectively capture utilisation of mobility devices or equipment across distances (5, 50 and 500 m) or settings (therapy, household, neighbourhood, community) respectively, they do not capture patient reported variables such as pain or fatigue that may impact upon mobility performance across different settings (home, school/work and community). Evaluation of patient reported outcomes is essential when considering the overall health status of an individual [22], and while the recently developed PROMIS tools provide questions regarding mobility, pain and fatigue [23], the impact of symptoms on mobility in different settings is not covered within the current item banks.

This study is proposing a functional mobility scale specifically for individuals with skeletal dysplasia. The purpose of this scale is to assist clinicians to objectively classify functional mobility, reflecting varying abilities across different real-world environment constructs (home, school/work, community), as well as capturing patient reported symptomatology such as pain or fatigue associated with this. The Screening Tool for Everyday Mobility and Symptoms (STEMS) considers functional mobility across different settings, and with varying types of mobility aides, in a similar method to FMS. However, the proposed scale also records and reflects the presence of symptoms such as pain and/or fatigue that impact activity levels for individuals with skeletal dysplasia. This allows clinicians to record the actual performance for the individual and capture patient-reported consequences. The recording of clinically relevant secondary outcomes such as pain and fatigue related to functional mobility level allows health care workers to assess and record changes that occur through provision of environmental modifications, equipment, rehabilitation programmes or medical and surgical interventions. The opportunity to provide a standardised screening tool to monitor everyday mobility and symptoms has potential for use as an efficacy end-point in future long-term clinical drug trials [24].

The aim of this study was to validate the screening tool (Screening Tool for Everyday Mobility and Symptoms; STEMS) by comparing the performance of a group of children and adults with skeletal dysplasia using STEMS with a contemporaneous mobility assessment of the same individuals using FMS and 6 min Walk Test (6MWT).

## Method

### Study design and participants

This cross-sectional study involved 150 Australian children and adults with any form of skeletal dysplasia aged between 5 and 75 years (recruited from August 2017 to November 2019). Individuals with any additional neurological (e.g. epilepsy, history of brain injury or cerebral palsy), musculoskeletal (e.g. scoliosis, recent fracture), respiratory or behavioural problems, which were not related to skeletal dysplasia, were excluded as these conditions may have impacted upon functional mobility level. Participants were identified and recruited across Australia at patient organisation conferences and the Kids Rehab Complex Musculoskeletal Clinic at The Children's Hospital at Westmead, New South Wales, the Rehabilitation Clinic at Queensland Children's Hospital Queensland, and the Bone Dysplasia Clinic at Victorian Clinical Genetics Services, Victoria. Ethical approval was obtained through the Sydney Children’s Hospitals Network (HREC/16/SCHN/221) and Macquarie University (Reference no. 5201700121) Human Research Ethics Committees. Written informed consent was obtained from individuals or, if under the age of 18 years, their parents/guardians.

### Measurement tools

Functional mobility was screened through the newly developed Screening Tool for Everyday Mobility and Symptoms (STEMS) (Additional file 1). The STEMS was developed using a reference group of expert clinicians and researchers working with individuals with skeletal dysplasia. Expert clinicians report that mobility aides are often supplied to individuals with skeletal dysplasia to maximise participation even when an individual has the physical capacity to mobilise without the aid. For example, an individual with short stature may require a powered device to maintain speed when mobilising with normal statured peers, or an individual with bone fragility may use a mobility aid to maintain safety when in crowded environments. Hence the scoring of an individual’s mobility based solely on the aid used does not necessarily reflect their physical capacity. Furthermore, symptoms are commonly reported to impact on the daily function of individuals with skeletal dysplasia, and may also result in the prescription of a mobility aide in attempts to minimise these [25, 26]. Therefore, the scoring system was developed to reflect both the usage of a mobility aide (reported by numbers) and the commonly reported clinical symptoms related to mobility levels (reported by letters). The STEMS considers mobility across different environments (home, school/work and community) and records usage of varying types of walking devices. The proposed STEMS also reflects pain and/or fatigue that may influence activity levels of individuals with skeletal dysplasia at the end of the day. Assessors identify the use of mobility aides across each of the three settings (home, school/work and community) and record this as a numerical value (1–5) (Score 1 = independent on all surfaces including stairs, 2 = use of sticks, 3 = use of crutches, 4 = use of wheeled walking device, 5 = use of wheelchair or mobility scooter) (Table 1). Assessors then record the impact that mobility has on function at the end of the day by including patient reported symptoms. Each symptom was assigned a letter; A (reflecting nil pain or fatigue), B1 (reflecting pain only), B2 (reflecting fatigue only) or C (reflecting both pain and fatigue). This is completed for each of the three environments so the individual receives three scores. The number reflects the use of mobility aides and the letter reflects the symptoms. e.g. 1A, 1B1, 1C. This example represents the situation where an individual mobilises at home with no aide and no pain or fatigue altering function at the end of the day (1A) but reports pain  after mobilising at work/school without use of aides (1B1) and both pain and fatigue after mobilising in the community without use of aides (1C).

**Table 1:** Comparison of scoring system between FMS and STEMS

	FMS	STEMS—aide	STEMS—symptoms
1	Uses wheelchair, stroller or buggy	Mobilises independently—no aide	A—Nil pain or fatigue altering activity
2	Uses K-walker or other walking frame	Mobilises—uses stick(s)	
3	Uses two crutches	Mobilises—uses crutches	B1—Pain limiting activity B2—Fatigue limiting activity
4	Uses one crutch or two sticks	Mobilises—uses wheeled walking device	
5	Independent on level surfaces	Wheelchair or power mobility scooter	C—Pain and fatigue limiting activity
6	Independent on all surfaces	N/A	

Participants were also assessed using the FMS for cerebral palsy, which was developed in 2004 to describe functional mobility in children with cerebral palsy. The FMS has been formally evaluated in terms of validity and reliability [20, 27, 28]. The FMS records the individual’s ability to walk a distance of 5, 50 and 500 m and uses a six point rating scale to record the use of assistive devices ranging from a score of 1 reflecting use of a wheelchair through to a score of 6 reflecting independent mobility on all surfaces (Table 1). The FMS was administered through interview by one of the researchers. Participants also completed the 6 min Walk Test (6MWT), a self-paced, standardized test of endurance and walking ability which has been demonstrated to be highly reliable and valid in both paediatric and adult populations [29–32]. Participants completed this test with or without sticks, crutches or a wheeled walking device. Wheelchair use was not allowed during the 6MWT.

### Procedure

Following collection of demographic data, including age, gender, diagnosis and any comorbidities, leg length was measured from the anterior superior iliac spine to the medial malleoli bilaterally. Standing height and weight was also recorded. The STEMS, FMS, and the 6MWT were completed by each participant under guidance from an experienced member of the research team. Parents/ guardians of younger children assisted in the completion of the STEMS and FMS. No direct age cut-off was used for parent or child-report, as a pragmatic approach was taken whereby the child was asked first and parent/guardian only contributed if the child was unable to answer.

### Data analysis

Analysis was conducted using SPSS for Mac (Version 26, Chicago, IL, USA). Data was examined visually and confirmed to be normally distributed, with the mean, standard deviations and frequencies calculated to describe the participant population. To account for differences in stature and proportions between individuals with skeletal dysplasia, all 6MWT total distances were normalised by dividing the total 6MWT distance by the average of the participants left and right leg length (metres).

To determine the usefulness of the STEMS to record an individuals’ functional mobility, including usage of mobility devices across three primary environments (home, school/work, community), the association between the STEMS and the FMS and the normalised 6MWT was undertaken using Pearson’s correlation coefficient. The correlation between the STEMS and each of the comparison tests (FMS and 6MWT) was defined as large if the correlation was 0.5–1.0, medium if 0.3—0.49 and small if 0.1–0.29 [33].

To identify and record the impact of additional symptomatology (pain and/or fatigue) on participation performance, two one-way between-groups analyses of variance (ANOVA) were conducted to explore the impact of i) symptom classifications (A, B1, B2 and C), and ii) number of symptoms (no symptoms (A), one symptom (B1 or B2), 2 symptoms (C)) within each of the three environments, on the normalised 6MWT distance. Impact of symptoms was explored within the total group of individuals with skeletal dysplasia, and due to sufficient sample size, also within the achondroplasia diagnostic category. Effect sizes were calculated using eta squared with 0.01 considered a small effect, 0.04 a medium effect and 0.14 a large effect [34]. When significant differences were noted, post hoc comparisons were performed with the Tukey HSD test to determine whether a particular symptom, group, or number of symptoms significantly altered walking ability.

## Results

### Demographics and population description

One hundred and fifty individuals with varying forms of skeletal dysplasia from across Australia enrolled in the study. Efforts were made to recruit approximately equal numbers of males and females across each five year (paediatric/adolescent) and ten year (adult) age bracket. Eighty four individuals (36 males) were classified as children or adolescents aged between 5 and 20 years with the remaining sixty-six (21 males) classified as adults (> 20 years). Participants were classified into three main groups based on diagnosis (numbers). Seventy-six individuals (28 males) had achondroplasia, forty-two individuals (14 males) had osteogenesis imperfecta and thirty two individuals (15 males) reported another form of skeletal dysplasia (Table 2). The ‘other’ group included thirty two individuals with forms of skeletal dysplasia classified as; (1) conditions causing short limbs at birth/infancy (including Spondyloepiphyseal dysplasia congenita (SEDC), pseudoachondroplasia, Spondyloepimetaphyseal dysplasia-Strudwick (S’E’MD-Strudwick), Diastrophic Dysplasia); (2) conditions causing short stature in later childhood (Metaphyseal chondrodysplasia type Schmid (MCDS), Leri Weil Dysplasia); (3) conditions affecting epiphyses (Multiple epiphyseal dysplasia (MED); (4) disorders with short hands/feet (Geleophysic dysplasia) and v) disorders that cause small chest size (Ellis can Creveld, Jeune asphyxiating thoracic dysplasia) [1]. The age-sex distribution and diagnostic groupings are reported in Table 2 and the mean heights are reported in Table 3. Eighty percent of the cohort (45 males; 75 females) were considered as short statured (less than 3rd centile height).

**Table 2:** Demographics and skeletal dysplasia diagnosis by gender and age in adults and children/adolescents (n = 150)

	Child/adolescent						Adults							
	5–10 years		11–15 years		16–20 years		21–30 years		31–40 years		41–50 years		> 51 years	
	M	F	M	F	M	F	M	F	M	F	M	F	M	F
**Achondroplasia**	15	15	4	7	5	5	1	5	0	6	3	5	0	5
**Osteogenesis imperfecta**	4	2	0	6	3	2	3	1	2	8	2	4	0	5
SEDC	0	2	1	0	1	1	2	1	0	0	2	0	0	1
Cartilage hair hypoplasia	0	1	0	0	0	0	0	0	1	0	0	0	0	0
Pseudochondroplasia	1	0	0	0	0	0	0	0	0	0	0	0	1	1
Hypochondroplasia	0	0	0	0	0	0	0	1	1	0	0	0	0	0
Primordial dwarfism	0	1	0	0	0	0	0	0	1	0	0	0	0	0
S’E’MD-Strudwick	0	1	1	0	0	0	0	0	0	0	1	0	0	0
Diastrophic dysplasia	0	2	0	0	0	0	0	0	0	0	0	0	0	0
Metaphysealchondro- dysplasia Schmid	0	0	0	1	0	0	0	0	0	0	0	0	0	0
Leri-Weil dysplasia	0	0	0	1	0	0	0	0	0	0	0	0	0	0
Multiple epiphyseal dysplasia	0	1	1	0	0	0	0	0	0	0	0	0	0	0
Geleophysic dysplasia	0	0	0	0	0	0	0	0	1	0	0	0	0	0
Jeune syndrome	0	0	0	0	0	0	0	0	0	1	0	0	0	0
Ellis-van Creveld syndrome	0	0	0	0	0	0	0	0	0	0	0	1	0	0
**Other—total**	1	8	3	2	1	1	2	2	4	1	3	1	1	2
**Total**	20	25	7	15	9	8	6	8	6	15	8	10	1	12

**Table 3:** Heights and deviations by skeletal dysplasia diagnosis for adults and children/adolescents

Dysplasia		Height in cm (range (mean ± SD))	
	Total N	Adult	Child/adolescent
Total	150	84.8–176.3 (133.8 ± 19.9)	77.8–175 (115.9 ± 22)
Achondroplasia	76	110–135 (125 ± 6.2)	83–149.9 (107.1 ± 14.8)
Osteogenesis imperfecta	42	84.8–176.3 (145.4 ± 25.1)	114.4–175 (142.5 ± 15.8)
Other	32	111–170 (128.9 ± 16)	77.8–154.5 (115.8 ± 24.6)

The FMS assessment [20] was used to describe functional mobility both for the total group and for each of the three major groupings (achondroplasia, osteogenesis imperfecta and Other) (Table 4). Across the total cohort there was a decrease in the number of 5/6 ratings (independent without walking aides) across 5, 50 and 500 m with a corresponding increase in the number of rating 1 (use of wheelchair, stroller or buggy) scores across the three distances. One fifth of the total cohort reported using a wheelchair or power mobility scooter over a distance of 500 m with 68% of wheelchair users being children.

**Table 4:** Functional mobility scale for study cohort

	Rating	Total (n/%)	Achondroplasia (n/%)	Osteogenesis imperfecta (n/%)	Other (n/%)
FMS-5	1	6 (4)	0 (0)	5 (11.9)	1 (3.1)
	2	2(1.3)	0 (0)	1 (2.4)	1 (3.1)
	3	2 (1.3)	0 (0)	1 (2.4)	1 (3.1)
	4	2 (1.3)	1 (1.3)	0 (0)	1 (3.1)
	5	12 (8)	6 (7.9)	5 (11.9)	1 (3.1)
	6	126 (84)	69 (90.8)	30 (71.4)	27 (84.4)
FMS-50	1	13 (8.7)	1 (1.3)	9 (21.4)	3 (9.4)
	2	1 (0.7)	0 (0)	0 (0)	1 (3.1)
	3	3 (2)	0 (0)	2 (4.8)	1 (3.1)
	4	5 (3.3)	1 (1.3)	2 (4.8)	2 (6.3)
	5	9 (6)	6 (7.9)	2 (4.8)	1 (3.1)
	6	119 (79.3)	68 (89.5)	27 (64.3)	24 (75)
FMS- 500	1	31(20.7)	8 (10.5)	12 (28.6)	11 (34.4)
	2	2 (1.3)	2 (2.6)	0 (0)	0 (0)
	3	5 (3.3)	1 (1.3)	3 (7.1)	1 (3.1)
	4	4 (2.7)	0 (0)	4 (9.5)	0 (0)
	5	4 (2.7)	2 (2.6)	1 (2.4)	1 (3.1)
	6	104 (69.3)	63 (82.9)	22 (52.4)	19 (59.4)

The 6MWT was not attempted by 5 individuals, who were unable to weightbear beyond standing transfers, all of whom had OI. The mean distance covered by the remaining 145 individuals was 414 m (range 20–705, SD 121 m; normalised 6MWT mean = 757, range 23–1347, SD 234 m). Nine participants including eight with OI and one with pseudoachondroplasia used a walking aide during the 6MWT.

### Correlations of the STEMS with FMS and 6 MWT

Using Pearson's rank correlations, the walking aids used across STEMS environment category demonstrated large significant negative correlations with the FMS tool: (1) STEMS—Home and FMS—5 m (r = − 0.968, p < 0.001); (2) STEMS—School/work and FMS—50 m (r = − 0.983, p < 0.001) and (3) STEMS—Community and FMS—500 m (r = − 0.994, p < 0.001). There was a medium negative significant correlation between the STEMS setting categories and the normalised 6MWT (r = − 0.323—-0.394, all p < 0.001).

### Symptomatology

**Fig. 1:**
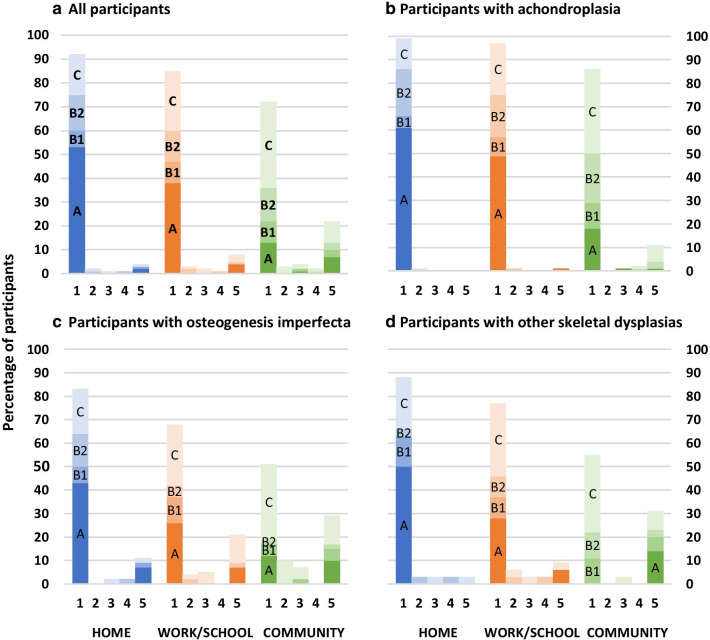
STEMS mobility aide and symptom classifications of all participants and participants within each diagnostic category, across different environments

Across the total cohort, there was a decrease in the number of participants reporting no symptoms (Score A) across home, school/work and the community with an increase in the number of participants reporting one symptom (either pain (B1) or fatigue (B2)) and two symptoms (pain and fatigue (C)) across each of the three environments (Fig. 1). Twenty percent of the total group reported symptoms of pain and fatigue that altered function associated with mobility around the home, rising to approximately one half indicating both pain and fatigue influencing function at the end of day associated with mobility around the community. The percentage of individuals reporting either pain or pain and fatigue increased from 28% within the home environment, to 42% within the school/work setting, and peaked at 62.6% resulting from community mobility. Across the diagnostic groups individually, individuals reporting both pain and fatigue in the community encompassed 44% of all individuals with achondroplasia, 81% of all individuals with OI and 38% of individuals with other skeletal dysplasia. Furthermore, 62% of the OI cohort reported two symptoms (Score C) compared with 50% of the Other group and 43% of the achondroplasia group (Fig. 1) when considering mobility within the community.

STEMS mobility aide and symptom classifications of all participants and participants within each diagnostic category, across different environments.

**Fig. 2:**
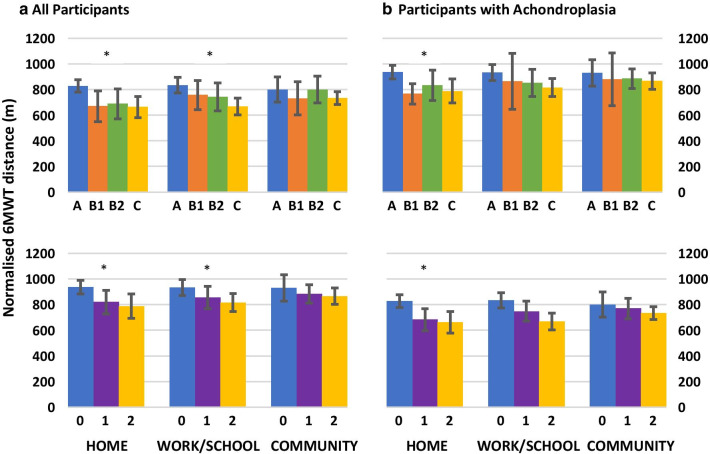
Normalised six minute walk distances covered by individuals based on symptomatology. The upper row in the panel demonstrates the type of symptom based on STEMS classification. The lower row indicates the number of symptoms. *indicate significant at p < 0.05 level

When considering the overall cohort, there was a moderate statistically significant difference in the normalised 6MWT distances achieved by individuals reporting zero, one or two symptoms (F(2,142) = 8.25, eta^2^ = 0.1, p = 0.001) and within each of the symptom classifications (F(3,141) = 5.48, eta^2^ = 0.1, p < 0.001) when mobilising around the home (Fig. 2). Post-hoc analysis demonstrated that individuals presenting with no symptoms (A) walked a significantly larger normalised 6MWT distance than individuals reporting one (mean difference 145.3 m, 95% CI 37.9–252.8 m) or two (mean difference 164.4 m, 95% CI 47.3–281.4 m) symptoms. Fatigue (mean difference 139.6 m, 95% CI 3.4–275.9 m) or pain and fatigue (mean difference 164.4 m, 95% CI 47.3–281.4 m) but not pain alone (mean difference 157.2 m, 95% CI − 26.8–341.2 m) reported to limit their ability around the home, significantly reducing the walking distances achieved on the 6MWT.

Moderate significant differences were also demonstrated when considering the limiting symptoms in the school/work environment and the normalised 6MWT distances (F = 4,53–6.89, eta^2^ = 0.09, p = 0.001–0.004). Individuals reporting pain and fatigue (mean difference 165.5 m, 95% CI 48.8–282.2 m), walked significantly lower distances than individuals reporting no symptoms.

No significant differences in the 6MWT distances were achieved between individuals reporting each of the different symptom classifications (F(3,141) = 0.86, *n* = 0.02, p = 0.46) as well as numbers of symptoms (F(2,142) = 0.64, eta^2^ = 0.12, p = 0.42) limiting community mobility.

The achondroplasia cohort also demonstrated a moderate statistically significant difference in the normalised 6MWT distances achieved by individuals reporting zero, one or two symptoms (F(2,73) = 4.96, *n* = 0.12, p = 0.01) and within each of the symptom classifications (F(3, 72) = 3.44, *n* = 0.12, p = 0.02) when mobilising around the home (Fig. 2). Post-hoc analysis also demonstrated that individuals presenting with no symptoms (A) walked a significantly larger normalised 6MWT distance than individuals reporting one (mean difference 116.3 m, 95% CI 2.1–230.6 m) or two (mean difference 148 m, 95% CI 7.4–288.6 m) symptoms limiting their ability to mobilise around the home. However, post-hoc testing demonstrated no significant differences between the symptom classifications. There were no significant differences in the normalised 6MWT distance and reporting of symptoms limiting the work/school and community setting (F = 0.42–6.36, eta^2^ = 0.02–0.07, p = 0.06–0.74).

Normalised six minute walk distances covered by individuals based on symptomatology.

## Discussion

This study describes a new screening tool (acronym “STEMS”) that allows clinicians to record mobility aide usage and patient-reported pain and fatigue while mobilising in three environments encountered in everyday life. There are currently no other tools for this for individuals with skeletal dysplasia. Clinical management focuses on maximising functional capacity which often includes recommendations for mobility aides and minimising the impact of symptoms on daily mobility. The use of the STEMS in clinical practice will therefore provide an efficient and valid way in which to capture actual day to day baseline performance and any change, which is important to both clinicians and patients alike.

The new STEMS was highly correlated with the mobility aide usage reported by the FMS tool. However, unlike the distance requirements (5/50/500 m) reported in the FMS, the STEMS screens an individual’s performance across three environments (home, school/work and community). In doing so, the STEMS considers how the combination of distance, speed of mobility, type of terrain and mobility demands may influence the need for a mobility aide. Furthermore, the STEMS captures patient reported symptoms such as pain and/or fatigue that may impact upon mobility performance across differing environments. This is likely reflected by the significant but lower correlation between the STEMS mobility aide categories and normalised 6MWT. The 6MWT, while a gold standard assessment in many clinical trials, is a measure of performance in a controlled environment under test conditions, not actual performance over time in a real world setting. Assessing capacity over a six minute period at a single point in time may also not have been sufficiently sensitive or challenging to differentiate variations in walking distance that may become more evident across greater distances and time and vary day to day as symptoms vary.

In accordance with previous research, this study identified pain as highly prevalent in individuals with short stature [18, 19]. While the numbers of people reporting fatigue remained stable across the different environments, there was an increase in the numbers of people reporting either pain or pain and fatigue as the environment became more challenging. This suggests that pain alone is an issue but that pain and fatigue combined is the greater challenge for individuals with skeletal dysplasia. This is the first report that considers fatigue as a significant factor impacting on mobility of individuals with skeletal dysplasia of both short and typical stature.

Almost two thirds of individuals in the current cohort reported pain or pain and fatigue associated with mobility in the community. These results are slightly less than the 75% of individuals noting pain reported by Dhiman et al. [19] in a recent study of pain in adults with skeletal dysplasia. Dhiman’s group described the severity and frequency of pain but did not link this to mobility or functional factors and did not include fatigue in their assessment. A much higher proportion of adults older than 51 years (44% in Dhiman’s study compared with 10% in the current study) may account for differences between the groups. However, despite over 62% percent of participants in this current study reporting pain alone or pain and fatigue when mobilising around the community, only twenty percent of individuals were scored as using a wheelchair or scooter across this environment. The majority of those utilising a wheelchair or power mobility scooter were children (68%), suggesting that a high proportion of adults with a form of skeletal dysplasia reporting pain or pain and fatigue within the community are not utilising a mobility device that may assist with overall symptom reduction. This is commonly seen in the paediatric population group where children will frequently use scooters to mobilise around the community as a mobility access strategy. The distinction between whether an aide was used to increase participation or ease symptomatology was not ascertained as part of this trial. Use of the STEMS would allow clinicians to more accurately record symptoms associated with everyday mobility across different distances and environments, and evaluate changes associated with intervention, such as provision of mobility aide or surgery. The STEMS would also allow individuals and clinicians to more accurately reflect changes associated with aging or disease process and progression.

Significant associations were identified between number of symptoms and type of symptoms reported by both the combined and the achondroplasia groups when mobilising around the home and school/work, and distances walked on the normalised 6MWT distance. However, there were no significant differences observed for either the combined or achondroplasia specific group linking number or type of symptoms across the community setting and distance covered in the 6MWT. The recognition that people reporting symptoms of pain, fatigue or both associated with mobility across the home and school/work environment, tended to mobilise shorter distances on the 6MWT suggests that more significant body structure and function impairments or disease process may be present in these individuals.

A limitation to this study relates to the sample size for the total cohort and individual diagnostic groupings. Information was provided to both adults and children with differing forms of skeletal dysplasia through clinical settings and through liaison with patient support groups such as the Short Statured People’s Association of Australia and the Osteogenesis Imperfecta Society of Australia. However, due to the relatively low numbers of individuals linked with patient support groups, it proved more challenging to recruit adults whilst children were more easily accessed through specialist tertiary public hospital clinics. Despite these challenges, over 44% of participants in this study were aged over 21 years, providing some access to information and any differing challenges reported by adults related to mobility and symptoms. To ensure representation across a variety of diagnoses and phenotypes, efforts were made to ensure that people with a variety of differing skeletal dysplasia forms were included in the total cohort.

The STEMS appears to be a useful new clinical tool to screen everyday mobility and associated symptoms of pain and/or fatigue in adults and children with a skeletal dysplasia. Inclusion of this tool will provide clinicians, families and individuals with the ability to record use of different mobility devices and identify symptoms associated with mobility across different environments. This will also allow for the monitoring of changes associated with aging or implementation of different or novel treatments, including drug therapies, which are emerging at a rapid pace for skeletal dysplasia [35]. A greater understanding of the links between functional mobility and associated symptomatology will be gained through ongoing research in this area. Further research is also necessary to understand the relative contribution that varying biomechanical, psychological or anthropometric features may have upon functional mobility and associated symptoms. Having a clearer understanding about links between anatomical impairments and presentations, and mobility will assist clinicians to better identify those individuals who would benefit from monitoring and intervention.

## Conclusions

The Screening Tool for Everyday Mobility and Symptoms (STEMS) is a useful new tool to identify and record mobility aide use and self-reported symptoms across three environmental settings for adults and children with skeletal dysplasia. The STEMS may assist clinicians to monitor individuals for changes in functional mobility and symptoms over time, identify individuals who are functioning poorly compared to peers and need further assessment and to measure effectiveness of treatment interventions in both clinical and research settings.

## Supplementary Information

**Additional file 1.** Screening Tool for Everyday Mobility and Symptoms (STEMS).

## Data Availability

The datasets generated and/or analysed during the current study are not publicly available due to restrictions imposed by the Ethics approval, but are available from the corresponding author on reasonable request.

## Abbreviations

STEMS: Screening Tool for Everyday Mobility and Symptoms
FMS: Functional Mobility Scale
6MWT: Six Minute Walk Test
OI: Osteogenesis imperfecta
Achon: Achondroplasia
SEDC: Spondyloepiphyseal dysplasia congenital
SMD: Spondyloepimetaphyseal dysplasia
MED: Multiple epiphyseal dysplasia
