# Supplementary material for: Development of the Screening Tool for Everyday Mobility and Symptoms (STEMS) for skeletal dysplasia
Source: Orphanet J Rare Dis. 2021 Jan 21;16:40. doi: 10.1186/s13023-021-01681-z (PMC7818550; doi:10.1186/s13023-021-01681-z)
Supplement: Supplementary file 1 — Additional file 1. Screening Tool for Everyday Mobility and Symptoms (STEMS). [file 13023_2021_1681_MOESM1_ESM.docx]

**Additional File 1: Screening Tool for Everyday Mobility and Symptoms (STEMS)**

| **Score 1** | **A** | Mobilises independently around this environment without requiring mobility aide. No activity limiting pain or fatigue reported. |
| --- | --- | --- |
|  | **B1** | Mobilises independently around this environment without requiring mobility aide. Reports pain that alters activity levels at end of day. |
|  | **B2** | Mobilises independently around this environment without requiring mobility aide. Reports fatigue that alters activity levels at end of day. |
|  | **C** | Mobilises independently around this environment without requiring mobility aide. Reports both pain and fatigue that alters activity levels at end of day. |
| **Score 2** | **A** | Mobilises around this environment using stick(s). No additional activity limiting pain or fatigue reported. |
|  | **B1** | Mobilises around this environment using stick(s). Reports pain that alters activity levels at end of day. |
|  | **B2** | Mobilises around this environment using stick(s). Reports fatigue that alters activity levels at end of day. |
|  | **C** | Mobilises around this environment using stick(s). Reports both pain and fatigue that alters activity levels at end of day. |
| **Score 3** | **A** | Mobilises around this environment using crutch(es). No additional activity limiting pain or fatigue reported. |
|  | **B1** | Mobilises around this environment using crutch(es). Reports pain that alters activity levels at end of day. |
|  | **B2** | Mobilises around this environment using crutch(es). Reports fatigue that alters activity levels at end of day. |
|  | **C** | Mobilises around this environment using crutch(es). Reports both pain and fatigue that alters activity levels at end of day. |
| **Score 4** | **A** | Mobilises around this environment using wheeled walking device. No additional activity limiting pain or fatigue reported. |
|  | **B1** | Mobilises around this environment using wheeled walking device. Reports pain that alters activity levels at end of day. |
|  | **B2** | Mobilises around this environment using wheeled walking device. Reports fatigue that alters activity levels at end of day. |
|  | **C** | Mobilises around this environment using wheeled walking device. Reports both pain and fatigue that alters activity levels at end of day. |
| **Score 5** | **A** | Mobilises around this environment using wheelchair or power mobility scooter. No additional activity limiting pain or fatigue reported. |
|  | **B1** | Mobilises around this environment using wheelchair or power mobility scooter. Reports pain that alters activity levels at end of day. |
|  | **B2** | Mobilises around this environment using wheelchair or power mobility scooter. Reports fatigue that alters activity levels at end of day. |
|  | **C** | Mobilises around this environment using wheelchair or power mobility scooter. Reports both pain and fatigue that alters activity levels at end of day. |
